# Influence of microbiota inoculum as a substitute for antibiotic growth promoter during the initial laying phase on productivity performance, egg quality, and the morphology of reproductive organs in laying hens

**DOI:** 10.14202/vetworld.2023.1461-1467

**Published:** 2023-07-19

**Authors:** Bodhi Agustono, Sunaryo Hadi Warsito, Maya Nurwartanti Yunita, Widya Paramita Lokapirnasari, Sri Hidanah, Emy Koestanti Sabdoningrum, Mohammad Anam Al-Arif, Mirni Lamid, Gandul Atik Yuliani, Shekhar Chhetri, Sarasati Windria

**Affiliations:** 1 Division of Animal Husbandry, School of Health and Life Sciences (SIKIA), Surabaya 60115, Indonesia; 2 Department of Veterinary Science, Division of Animal Husbandry, Faculty of Veterinary Medicine, Universitas Airlangga, Surabaya 60115, Indonesia; 3 Division of Pathology Veterinary, School of Health and Life Sciences (SIKIA), Universitas Airlangga, Surabaya 60115, Indonesia; 4 Department of Veterinary Science, Division of Basic Veterinary, Faculty of Veterinary Medicine, Universitas Airlangga, Surabaya 60115, Indonesia; 5 Department of Animal Science, Royal University of Bhutan, Thimphu, Bhutan; 6 Department of Biomedical Science, Faculty of Medicine, Universitas Padjajaran, Bandung, Indonesia

**Keywords:** external quality eggs, good health, growth performance, internal quality eggs, probiotics, reproductive organs

## Abstract

**Background and Aim:**

Antibiotics that increase growth have long been employed as a component of chicken growth. Long-term, unchecked usage may lead to microbial imbalance, resistance, and immune system suppression. Probiotics are a suitable and secure feed additive that may be provided as a solution. The objective of this research was to ascertain the effects of dietary multistrain probiotics (*Lactobacillus acidophilus*, *Bifidobacterium* spp., and *Lactobacillus plantarum*) on the morphology (length and weight) of reproductive organs and productivity performance of laying hens during the early stage of laying.

**Materials and Methods:**

One hundred ISA Brown commercial layer chicks of the same body weight (BW) that were 5 days old were divided into five treatments, each with four replicates and four chicks in each duplicate. There were five different dietary interventions: (T1) 100% base feed; (T2) base feed with 2.5 g of antibiotic growth promoter/kg feed; (T3) base feed plus probiotics; (T4) base feed at 1 mL/kg with probiotics; and (T5) base feed with probiotics, 3 mL/kg feed, 5 mL/kg of feed. The parameters observed were performance, internal and exterior egg quality, and the morphology (length and weight) of laying hens’ reproductive organs.

**Results:**

Probiotic supplementation (*L. acidophilus*, *Bifidobacterium*, and *L. plantarum*) significantly affected the BW, feed intake, egg weight, yolk index, albumin index, Haugh unit, egg height, egg width, and morphology (length and weight) of laying hens’ reproductive organs compared to the control group (basic feed). In addition, there was no discernible difference between treatment groups in theeggshell weight and thickness variables across all treatment groups.

**Conclusion:**

When laying hens were between 17 and 21 weeks old, during the early laying period, microbiota inoculum supplements (*L. acidophilus*, *Bifidobacterium*, and *L. plantarum*) increased growth, the quality of the internal and external layers’ eggs, and the morphology of the laying hens’ reproductive organs.

## Introduction

Antibiotic growth promoters (AGPs), or AGPs, have been increasingly employed in recent years to boost cattle production and growth [1]. Intensive management of chickens may encourage higher output and aid in the fight against illness [2]. Long-term and uncontrolled usage may change the intestinal bacterial population, affecting the digestive system, metabolic functions, and nutritional absorption [3]. In addition, antibiotics impact the immune system by modifying the bacterial ecology or the immunological response [4].

Several studies have shown chicken and its products to be reservoirs and potential sources of antibiotic-resistant *Salmonella* strains [5]. Under the present antimicrobial resistance (AMR) scenario, the Indonesian government has officially prohibited the use of AGPs as feed additives [6]. Preventing the use of AGP, making alternative materials a solution that can still increase production, and protecting animal health by preventing the formation of hazardous residues on its surface [7]. One way to deal with this problem is to use probiotics as an alternative feed additive.

Probiotics have been shown to enhance gut health, stabilize gut flora, and decrease pathogen colonization, making them an appealing replacement for antibiotics [8]. *Bifidobacterium* spp., Dan *Lactobacillus acidophilus*, and *Lactobacillus plantarum* are a few probiotic isolates that may be employed [9, 10]. Due to their impact on nutritional absorption, adding these bacteria may impact the villi in the gut [11]. Feeding laying hens according to their nutritional needs during the prelayer period can maintain and increase egg production until peak egg production [12]. In addition, 20 weeks before puberty, the development of the reproductive system reaches its pinnacle, and throughout the production era, organ growth is mostly constant. Lack of the nutrients that layer hens need may harm and damage the reproductive system, reducing egg production [13]. According to previous studies, probiotic dietary supplementation enhances egg production and improves chicken performance, feed conversion efficiency, and eggshell quality [14, 15]. In addition, probiotics have been shown to enhance intestinal goblet cells, promote intestinal T-cell immunity, and control the colonization of symbiotic bacteria [16, 17]. Because probiotics regulate intestinal microbiota, increase the availability of nutrients in poultry, boost body immunity, and may enhance laying hens’ blood profiles, adding them to the diet can be advantageous for poultry [16, 18]. The reduction in body weight (BW) variances until the first laying phase of laying hens during the pre-laying period will have an influence on the productivity of succeeding laying hens. These data give persuasive evidence that probiotics may enhance the growth rate of laying chickens at 17–21 weeks of age. This study aimed to determine the effects of dietary multistrain probiotics that are *L. acidophilus*, *Bifidobacterium* spp., and *L. plantarum* on the morphological characteristics (length and weight) of reproductive organs, productivity performance, and egg quality of laying hens during the early laying phase.

## Materials and Methods

### Ethical approval

The study was approved by the Ethical Committee of Universitas Airlangga, Indonesia, with number 15/HRECC.FODM/IX/2022.

### Study period and location

The research was conducted for 36 days (June–July 2022). Location of research conducted, namely, treatment of experimental animals in experimental animal cages at Airlangga University and measurement of productivity performance (growth of reproductive organs and pubertal characteristics and egg quality at puberty) at the Airlangga University Feed and Nutrition Laboratory.

### Experimental design

The experimental animal used in this study was an ISA Brown strain chicken aged 17 weeks with a BW of 1.5 kg (±0.5). The probiotic bacteria used included *L. acidophilus*, *Bifidobacterium*, and *L. plantarum*, with a concentration of 1.2 × 10^9^ colony-forming unit (CFU)/mL. The layer used is commercial feed with a crude protein content of 18%–19%.This study followed a completely randomized design consisting of five treatments, where each treatment consisted of four replications and each replication had five birds for a total of 100 animals. The treatments used in this study were five (T1–T5), consisting of (T1) 100% basal feed, (T2) basal feed + 2.5 g AGP/kg feed, (T3) basal feed + probiotics 1 mL/kg feed, (T4) basal feed + probiotics 3 mL/kg feed, and (T5) basal feed + probiotic 5 mL/kg feed.

### Data collection technique

Study topics included growth efficiency, reproductive system development, puberty traits, and the quality of the eggs produced throughout puberty.

### Eggs were weighed on a computerized scale

Feed intake (g) = Feed offered (g) – remaining feed (g) to acquire data on egg weight.

Health, mortality, feed consumption, laying efficiency, and egg quality assessments were made daily. Three healthy layers were chosen from each group replication at the conclusion of the experiment, when the 60 chicks were 22 weeks old (12 in each group), and they were then slaughtered. The organs were taken to calculate the weight of the corpse.

### Egg quality

Egg quality is divided into two perspectives, namely, external and internal. It can be assessed by assessing the eggshell, egg mass, egg height, and egg width. In contrast, the internal egg quality calculation can be done by measuring egg yolk height, egg yolk color, albumin thickness, egg white index (albumin index), egg yolk index, and the calculation of Haugh units (HU).

The egg yolk index was the ratio between the height and diameter of the yolk egg.

The egg white index was the measurement result of the albumin (thick albumin) using a caliper.

The calculation of the HU used the formula discovered by Haugh in 1937 [19].

### Reproductive organs

After necropsy, the reproductive organs of the birds were collected. Each organ was classified by length and weight. Digital calipers were used to measure follicles. The reproductive organs, including the ovaries, infundibulum, Magnum, isthmus, uterus, vagina, and cloaca, were dissected for layer-by-layer measurement. The ovaries contained numerous follicles, and the De Graaf follicles, mature for oviduct ovulation, were measured from the ostium to the cloaca. The infundibulum organs, with a funnel shape, were cut near the ovary. The length of the section of the infundibulum in this study was not measured due to unclear organ boundaries. The Magnum organs were distinguished from the isthmus by slicing them 35 cm before the isthmus. The isthmus organ was considered standard at a length of +10 cm. After the onset, the uterus exhibited a dark red color and creases [20].

### Statistical analysis

Data were collected and analyzed using the Statistical Package for the Social Sciences version 25.0 software (IBM Corp., NY, USA). The data obtained were tested by analysis of variance (ANOVA) with confidence (5%), and if there was a significant difference, then proceed with the distance test by Duncan test.

## Results

### Growth performance

The growth performance of laying hens was assessed based on variables such as BW, carcass weight, and feed consumption. In this study, the supplementation of probiotics (*L. acidophilus*, *Bifidobacterium*, and *L. plantarum*) at doses of 1, 3, and 5 mL/kg feed (T3, T4, and T5) resulted in an increase in the BW of laying hens compared to the control group (T1), as shown in Table-1. Furthermore, significant differences in feed consumption were observed between groups T2, T3, T4, and T5 compared to group T1 (Table-1). The results indicated similar feed consumption between the probiotic treatment group and the group receiving AGP, while the probiotic treatment showed higher feed efficiency. In contrast, the control group exhibited the lowest feed efficiency value.

**Table-1:** Growth performance of layer chicken fed different experimental diets during pre-layer period.

Variables	T1	T2	T3	T4	T5
Initial weight (g)	1441 ± 26.15^a^	1440 ± 12.4^a^	1446 ± 34.9^a^	1441 ± 13.7^a^	1445 ± 22.3^a^
Body weight (g)	1730.07 ± 29.59^a^	1750 ± 23.09^b^	1766 ± 31.70^b^	1770 ± 34.38^b^	1745.31 ± 35.4^b^
Feed consumption (g)	102.95 ± 3.5^a^	115.65 ± 3.8^b^	118.6 ± 3.8^b^	117.5 ± 5.5^b^	118.2 ± 3.8^b^
Carcass weight (g)	1136.82 ± 26.1^a^	1189.82 ± 29.1^b^	1205.64 ± 23.70^b^	1210.68 ± 21.8^b^	1199.02 ± 31.2^b^

^a, b, c^The different superscripts on the bar chart show significant differences (p<0.05). (T1) 100% basal feed, (T2) basal feed+2.5 g of AGP/kg feed, (T3) basal feed+probiotics 1 mL/kg feed, (T4) basal feed+probiotics 3 mL/kg feed, (T5) basal feed+probiotic 5 mL/kg feed. AGP=Antibiotic growth promoters

### Internal quality eggs

#### Yolk color score

In this investigation, supplementing laying hens’ diet with probiotics at doses of 1, 3, and 5 mL/kg (T3, T4, and T5) led to an increase in the weight of their eggs compared to treatment group T1 (Table-2).

**Table-2:** Reproduction organ weight, reproduction organ length (cm), internal egg quality, and external egg quality of laying hens in the early phase of laying.

Variables	T1	T2	T3	T4	T5
Reproduction organ weight (g)					
Vagina	4.66^a^ ± 0.77	4.92^a^ ± 0.75	5.92 ± 0.37	8.44^c^ ± 0.83	8.64^c^ ± 0.45
Uterus	11.50^a^ ± 0.85	14.20^b^ ± 0.78	15.42^c^ ± 0.21	15.92^c^ ± 0.67	16.12^c^ ± 0.35
Oviduct	94.69^a^ ± 3.24	94.79^a^ ± 3.12	104.05^b^ ± 1.34	107.34^c^ ± 2.49	108.98^c^ ± 1.96
Ovarium	37.48^a^ ± 1.63	42.66^b^ ± 1.39	43.34^b^ ± 3.62	53.92^c^ ± 3.50	56.46^c^ ± 2.01
Magnum	22.74^a^ ± 2.02	23.40^a^ ± 1.88	26.04^a^ ± 1.75	29.44^b^ ± 3.70	29.44^b^ ± 3.71
Cloaca	4.06^a^ ± 0.25	6.82^b^ ± 0.38	7.08^b^ ± 0.35	11.60^c^ ± 0.30	11.70^c^ ± 0.29
Isthmus	5.54^a^ ± 0.11	6.12^b^ ± 0.41	8.06^c^ ± 0.51	8.44^c^ ± 0.31	8.44^c^ ± 0.32
Follicle	9.10^a^ ± 0.51	9.10^a^ ± 0.54	11.20^b^ ± 0.44	11.78^b^ ± 0.42	11.87^b^ ± 0.31
Reproduction organ length (cm)					
Vagina	4.34^a^ ± 0.42	4.90^a^ ± 0.08	5.14^a^ ± 0.66	6.70^b^ ± 0.97	7.32^b^ ± 0.40
Uterus	5.00^a^ ± 0.61	5.50^a^ ± 0.50	5.68^a^ ± 0.66	7.68^b^ ± 0.78	7.78^b^ ± 0.30
Oviduct	75.34^a^ ± 1.78	77.7^ab^ ± 1.46	80.42^b^ ± 2.79	84.60^c^ ± 2.92	84.20^c^ ± 0.52
Ovarium	5.20^a^ ± 0.21	6.20^b^ ± 0.27	7.12^c^ ± 0.13	7.30^d^ ± 0.21	7.42^b^ ± 0.08
Magnum	28.40^a^ ± 1.91	30.80^a^ ± 2.16	33.80^b^ ± 0.83	37.00^c^ ± 1.54	36.60^c^ ± 1.08
Cloaca	4.56^a^ ± 0.37	4.88^ab^ ± 0.37	4.98^b^ ± 0.14	6.24^c^ ± 0.23	6.38^b^ ± 0.03
Isthmus	11.36^a^ ± 0.41	11.66^a^ ± 0.32	12.02^a^ ± 0.48	13.06^b^ ± 0.70	13.14^b^ ± 0.15
Follicle	5.60^a^ ± 0.41	6.50^b^ ± 0.50	7.40^c^ ± 0.41	8.00^c^ ± 0.70	8.10^c^ ± 0.41
Internal egg quality					
Yolk color score	6.70 ± 0.48^a^	6.75 ± 0.467^a^	7.80 ± 0.817^b^	8.40 ± 0.720^b, c^	8.823 ± 0.148^c^
Yolk index	0.488 ± 0.012^a^	0.486 ± 0.018^a^	0.502 ± 0.019^a^	0.523 ± 0.010^b^	0.527 ± 0.009^b^
Albumin index	0.164 ± 0.023^a^	0.1617 ± 0.024^a^	0.184 ± 0.034^a, b^	0.201 ± 0.017^b^	0.190 ± 0.003^a, b^
Haugh unit	97.32 ± 6.13^a^	97.60 ± 4.74^a^	102.27 ± 6.09^a, b^	106.80 ± 3.99^b^	104.09 ± 0.742^a, b^
External egg quality					
Egg weight (g)	49.98 ± 1.73^a^	51.11 ± 1.13^a, b^	52.42 ± 1.94^b, c^	53.54 ± 2.15^c^	53.51 ± 0.38^c^
Egg height (cm)	51.20 ± 0.49^a^	51.13 ± 1.05^a^	52.34 ± 0.38^b^	53.30 ± 0.60^c^	53.36 ± 0.12^c^
Egg width (cm)	40.24 ± 0.65^a^	40.17 ± 0.69^b^	41.18 ± 0.84^b^	41.58 ± 0.69^b^	40.95 ± 0.61^b, c^
Eggshell weight (g)	7.04 ± 0.15^a^	7.16 ± 0.20^a^	7.68 ± 0.24^b^	7.77 ± 0.25^b^	7.65 ± 0.014^b^
Eggshell thick (cm)	0.33 ± 0.013^a^	0.34 ± 0.017^a, b^	0.34 ± 0.006^b, c^	0.36 ± 0.012^c^	0.35 ± 0.002^b, c^

^a, b, c^The different superscripts on the bar chart show significant differences (p<0.05). (T1) 100% basal feed, (T2) basal feed+2.5 g of AGP/kg feed, (T3) basal feed+probiotics 1 mL/kg feed, (T4) basal feed+probiotics 3 mL/kg feed, (T5) basal feed+probiotic 5 mL/kg feed. AGP=Antibiotic growth promoters

#### Yolk index

In addition, for the yolk index, groups T1, T2, T3, T4, and T5 did not show significant differences between treatment groups (Table-2).

#### Albumin index

The difference was also seen in the albumin index variable, where the T4 group showed a significant difference from the T1 treatment group. In contrast, T2 and T3 did not differ significantly from T1 and T5.

#### Haugh unit

In this study, the calculation of the HU variable showed that the T4 group showed a significant difference from the T1 treatment group. While T2 and T3 did not significantly differ from T1 and T5, the results showed a high yolk color score variable where the T3, T4, and T5 groups showed a significant difference from the T1 treatment group. In contrast, T3 did not show a significant difference from T3.

### External quality egg

In this study, examination of the external quality of the observed eggs revealed egg weight, egg height, egg width, eggshell weight, and eggshell thickness. In general, probiotic supplementation (*L. acidophilus*, Bifidobacterium, and *L. plantarum*) at 1, 3, and 5 mL/kg feed (T3, T4, and T5) showed significant differences in egg height and width with other treatment groups. Furthermore, the variables of eggshell weight and thickness in basal feed (T1), basal feed + 2.5 g of AGP/kg feed (T2), and basal feed + probiotic (T3, T4, and T5) showed a significant difference (Table-2).

#### Reproductive organ

The study’s ANOVA on reproductive organ length demonstrated that the data were normally distributed and that the treatment group had significant differences (p < 0.05), so the Duncan test could be used. According to the vaginal organ length study, T1 was not substantially different from T2 and T3, while T1, T2, and T3 differed considerably from T4, T5, and T6.

The uterine organ data of the T1 treatment group showed significant differences with the basal feed + probiotic (T3, T4, and T5). Oviduct organs reveal that T1 treatment results are considerably different from T3, T4, and T5, but not from T2.

The Magnum organ study of basal feed (T1) and basal feed + 2.5 g of AGP/kg feed (T2) showed a significant difference with basal feed + probiotic (T3, T4, and T5) (Table-3). Cloaca organs were variable, and the T2, T3, T4, and T5 groups showed a significant difference from the T1 treatment group. Research on isthmus organ treatment found the T2, T3, T4, and T5 groups showed a significant difference from the T1 group. The follicular organ treatment found in the T3, T4, and T5 groups showed a significant difference from the T1 treatment group.

## Discussion

According to this study, compared to the T0 treatment group (treatment with basal feed and no feed additive), growth performance with the BW variable could be improved by giving a combination of feed additive inoculants of *L. acidophilus*, *L. plantarum*, and *Bifidobacterium* spp. Probiotics added to a base diet as a feed addition have been shown to increase BW in layer hens when compared to the control group [21]. There was no discernible change in the administration of probiotics, according to Chen *et al*. [22] and Junaid *et al*. [23]. Numerous metabolites and derivatives of the intestinal microbiota, such as short-chain fatty acids, lipopolysaccharides, secondary bile acids, trimethylamine, imidazole propionic acid, branch chain amino acids, and indole, can act as messengers to influence a range of aspects, including host energy homeostasis, obesity, appetite, blood sugar regulation, insulin sensitivity, inflammation, and endocrine regulation, as well as to control host metabolism [24]. Many probiotic strains are also added to the chicken feed to avoid dysbiosis or minimize the use of AGP, which is often used to reduce the load of pathogenic bacteria. The good bacteria continually work to prevent pathogen colonization of the gut and maintain gut maturation and integrity [25]. They also control the host’s immune response. According to Biswas *et al*. [26], adding probiotics might enhance growth performance. In addition, the specific role of the colonization of the gut microbiome, the development of the microbiota-gut-brain axis, or the development of the microbiota-gut-liver axis may constitute a substantial predictor of lifetime metabolic patterns [27]. In general, both internal (genetic) and external (environmental stress, food, farm cleanliness, and unidentified microbes) influences affect the ultimate look of chickens [28].

The egg weight variable findings revealed an increase in egg weight in the T4 and T5 treatment groups that received the probiotic feed additive compared to the T2 treatment group that received the AGP feed additive and the T1 control treatment group that did not receive the feed additive. This increase is consistent with several research findings that found a significant increase in egg weight variables in laying hens with *Lactobacillus* feed additive treatment [29, 30] from 20 to 68 weeks of age (Haddadin *et al*. [31]) compared to a control group that did not receive probiotics. Abdelqader *et al*. [32] also found that feeding supplements containing *Bacillus subtilis* inoculants increased egg weight. The same results were obtained in a study by Khan and Naz [33], where the treatment group showed a significant increase in egg weight and egg mass using commercial multistrain probiotics with 2 × 10^9^ CFU/g *L. plantarum*, *Lactobacillus bulgaricus*, *L. acidophilus*, *Lactobacillus* *rhamnosus*, *Bifidobacterium*, *Streptococcus thermophilus*, *Enterococcus faecium*, *A*. Based on the observed findings, it is conceivable that the inclusion of probiotic feed additives (*L. plantarum*, *L. acidophilus*, and *Bifidobacterium*) plays a function as a natural promoter of improved feed utilization in boosting egg production efficiency and egg weight. Internal (genetic) and extrinsic (environmental stress, food, farm cleanliness, and unidentified microbes) elements both impact the ultimate look of chickens [28].

In terms of egg external quality, there was a significant difference in the egg height and egg width variables in the probiotic group compared to the control group, but the eggshell weight and eggshell thickness variables were the same between the treatment groups. The findings showed compatibility with the length and diameter of eggs according to the Indonesian National Standard, which was 50 mm for the length and 35 mm for the diameter of the egg. The same study discovered that *L. plantarum* probiotic supplementation did not influence the eggs’ exterior quality [34]. Chicken eggshell thickness varies from 0.33 mm to 0.35 mm, with thinner shells having more holes and influencing shell weight [35]. The lack of an increase in eggshell weight and thickness in this study contradicts the findings of Sjofjan *et al*. [36], who found that eggshell and eggshell weight increased in the probiotic group compared to the control group, indicating that probiotics could increase acidic pH in the body. This may boost bone mineralization even further by boosting calcium and phosphorus absorption [37]. Following that, there was a rise in the number of probiotic and AGP treatment groups compared to the control group based on the findings of examining the internal quality of eggs, including the yolk color score, albumin index, and HU. Water was able to migrate from the intracellular medium to the extracellular medium due to less solute in the probiotic-supplemented treatment, improving the brightness of the yolk. Moisture levels on the gem’s surface increase due to the gem’s surface being more reflective of incident light throughout this process. More study is needed to understand the association between the activity of the gut microbial community and egg yolk color since the underlying mechanisms of action are not well understood [38]. The egg yolk index is computed by multiplying the egg yolk’s height by its diameter. The egg yolk index standard corresponding to Indonesian National Standard is divided into three categories: quality I is between 0.485 and 0.521, quality II is between 0.394 and 0.457, and quality III is between 0.330 and 0.393. According to the data, the egg yolk index (0.484–0.502) falls into quality group I. The egg white index is divided into three categories, according to Indonesian National Standards [39]: quality I (0.134–0.175), quality II (0.092–0.133), and quality III (0.05–0.091). The egg white index was classified as quality I (0.164–0.201) based on the findings. The egg white index is influenced by the protein level in the diet; if protein intake is high, ovomucin (egg white protein) is more desirable; the higher the score, According to Patterson and Burkholder [40], the efficacy of probiotics may be regulated by a variety of factors, such as application and delivery method, chicken strain, feed type, and probiotic concentration. Haugh unit is strongly connected to albumin dilution due to egg white ovomucin. Suparman *et al*. [41] determined that fresh chicken eggs had a HU of 100. According to SNI 2006, T1 and T2 had a HU value less than 100 but were nonetheless good-quality eggs, but T3, T4, and T5 had HU values more than 100 [42]. According to Carvalho [38], probiotics in chicken feed boost growth, reproduction, and egg quality. Neijat *et al*. [43] also observed an increase in yolk color score, HU, egg yolk index, and albumin index in laying hens treated with Bacillus inoculants. The gut environment also promotes enhanced egg internal and exterior quality measures by boosting mineral and other nutrient absorption of minerals and other nutrients [36, 43].

Compared to AGP and the control, probiotics boost digestion, increasing reproductive organ size and production. Probiotics enhance the acidity of the chicken’s digestive system. Probiotic bacteria may compete with and outcompete pathogenic bacteria in an acidic digestive tract [44]. According to Agustono *et al*. [45], intestinal nutrients are mostly required to sustain intestinal cell activity and regeneration. The small intestine digests protein and fat, which build tissue and promote cell proliferation. Gonadotropin-releasing hormone, which is released by proper food absorption, increases pituitary follicle stimulating hormone, luteinizing hormone (LH), and ovarian estrogen hormone. Follicle-stimulating hormone promotes ovarian and small follicle growth, maturation, and vascularization. Luteinizing hormone causes the ovum to develop. Follicular growth is the first step in the generation of estrogen. Serum and granulose cells stimulate estrogen release and the development of the reproductive tract [46]. Growing follicles in the active reproductive tract promote cell and tissue development, increasing the weight and length of the reproductive organs. Follicular growth decreases estrogen while increasing progesterone. Progesterone in the blood increases anterior pituitary LH secretion [47]. Increased LH promotes ovulation, which may lead to an increase in layer production.

## Conclusion

Probiotic supplementation (*L. acidophilus*, Bifidobacterium, and *L*. plantarum) during the early laying period at the age of 17–21 weeks increases growth, internal quality eggs, external quality eggs, and the morphology (length and weight) of the reproductive organs of laying hens. It is necessary to conduct detailed research on the activity of digestive enzymes and the level of digestibility of the nutrition of the feed given with the addition of probiotics or in combination with herbal extracts in laying hens of the ISA Brown line.

## Authors’ Contributions

BA, WPL, and SW: Supervised the study. BA, SHW, EKS, and MNY: Conducted the study. MAA: Statistical analysis and drafted the manuscript. BA, SHW, GAY, SC, and EKS: Data collection and drafted the manuscript. BA, SH, WPL, and ML: Prepared the treatment and revised the manuscript. All authors have read, reviewed, and approved the final manuscript.
